# Diagnostic accuracy of contrast enhanced ultrasound in patients with blunt abdominal trauma presenting to the emergency department: a systematic review and meta-analysis

**DOI:** 10.1038/s41598-017-04779-2

**Published:** 2017-06-30

**Authors:** Zhongheng Zhang, Yucai Hong, Ning Liu, Yuhao Chen

**Affiliations:** Department of emergency medicine, Sir Run-Run Shaw Hospital, Zhejiang University School of Medicine, Hangzhou, 310016 China

## Abstract

We aimed to investigate the diagnostic accuracy of contrast-enhanced ultrasound (CEUS) in evaluating blunt abdominal trauma for patients presenting to the emergency department. Electronic search of Scopus and Pubmed was performed from inception to September 2016. Human studies investigating the diagnostic accuracy of CEUS in identifying abdominal solid organ injuries were included. Risk of bias was assessed using the QUADAS tool. A total of 10 studies were included in the study and 9 of them were included for meta-analysis. The log(DOR) values ranged from 3.80 (95% CI: 2.81–4.79) to 8.52 (95% CI: 4.58–12.47) in component studies. The combined log(DOR) was 6.56 (95% CI: 5.66–7.45). The Cochran’s Q was 11.265 (p = 0.793 with 16 degrees of freedom), and the Higgins’ I^2^ was 0%. The CEUS had a sensitivity of 0.981 (95% CI: 0.868–0.950) and a false positive rate of 0.018 (95% CI: 0.010–0.032) for identifying parenchymal injuries, with an AUC of 0.984. CEUS performed at emergency department had good diagnostic accuracy in identifying abdominal solid organ injuries. CEUS can be recommended in monitoring solid organ injuries, especially for patients managed with non-operative strategy.

## Introduction

Trauma is one of the leading causes of death for teenagers and youngsters globally^1–3^. The key to the successful treatment of trauma is prompt identification of injured organs. If there is massive hemorrhage in the abdominal solid organs, urgent surgical intervention may help to save lives. For hemodynamically unstable patients, the identification of intraperitoneal fluid with ultrasound may warrant urgent laparotomy. The well-known FAST (focused assessment with sonography for trauma) algorithm is a technique developed for the assessment of a polytrauma with ultrasonography, especially in patients with compromised hemodynamic status^4, 5^. While ultrasound is valuable in the assessment of hemodynamically unstable patients with a huge hemoperitoneum, it is not sensitive enough to identify parenchymal injuries. Fortunately, novel ultrasound technique using second-generation ultrasound contrast agents, also known as contrast enhanced ultrasonography (CEUS), has been developed for use in polytrauma^6, 7^. Many clinical studies have been conducted to investigate the diagnostic performance of CEUS in detecting parenchymal injuries^8–11^. CEUS has also been recommended by guidelines for the evaluation of blunt abdominal trauma^12, 13^. However, there are conflicting results in these studies, and there may be difference in the diagnostic accuracy of CEUS for different solid organs (e.g. kidney, spleen and liver)^14–16^. Therefore, a systematic review of literature was performed in the present study, aiming to provide state-of-the-art evidence on the use of CEUS for patients presenting to the emergency department with blunt abdominal trauma.

## Methods

### Protocol and registration

The study protocol was registered at PROSPERO register (http://www.crd.york.ac.uk/PROSPERO), and the registration number was CRD42016048098.

### Eligibility criteria and study selection

Studies were considered eligible if they fulfilled the following criteria: 1) human studies investigating the diagnostic accuracy of CEUS for blunt abdominal trauma; 2) both prospective and retrospective studies were eligible; 3) the reference standard should be clearly defined. Studies were excluded when they met one of the following criteria: 1) animal experiments; 2) follow up studies using CEUS to investigate recovery of injured organs; 3) reviews and commentary; 4) duplicated reports; 5) studies investigating lumen organs such as gall bladder, intestine and bile duct; 6) non-trauma conditions (e.g. CEUS for solid organ tumors). There was no restriction on the year of publication and language.

Electronic databases of SCOPUS and Pubmed were searched from inception to September 2016. If there was missing information on quantitative data, we tried to contact the corresponding author for more information.

The electronic searching strategy consisted core terms related to Contrast-enhanced ultrasonography, trauma, and solid organs. Detailed searching strategies were shown in the appendix E1. There was no restriction on the language and publication years. Identified citations were firstly screened by their tiles and abstracts. Articles passed the initial screening were reviewed for the full text. Studies with data available on true positive (TP), false negative (FN), false positive (FP) and true negative (TN) were included for meta-analysis.

### Data collection process

Data from eligible studies were extracted based on a custom-made form. The extracted information included the name of the first author, publication year, study design, sample size, study population, solid organ, age of the study population, experience of the operator, type and dose of the ultrasound contrast agents, timing of CEUS and reference standard. Quantitative data on TP, FN, FP and TN were extracted from the original articles and collected using MS Excel-based form. Two authors independently extracted these data and disagreement was settled by a third opinion.

### Risk of bias

Risks of bias of individual studies were assessed using the QUADAS tool (Table 1). The tool consisted of 14 items, each of which should be scored as “yes”, “no” or “unclear” for an individual study. Item 1 was to assess spectrum bias in an individual study and it was scored “yes” if the individual study stated it included patients with blunt abdominal trauma. Item 2 was to assess whether the study explicitly define the inclusion and exclusion criteria. Because CT was considered to be able to correctly identify parenchymal injury, item 3 was scored “yes” if they used CT as reference standard. If an individual study described that CT scan was performed immediately after CEUS, the item 4 was scored as “yes”. If all participants received reference standard test for an individual study, item 5 was scored as “yes”. Item 6 was to exclude differential verification bias and this was “yes” if all participants received reference test irrespective of the CEUS results. Item 7 described the incorporation bias, and if review authors believed that interpretation of CT is independent of the CEUS results, item 7 was considered as “yes”. If sufficient details on how to perform CEUS and CT scan were described, item 8 and 9 should be “yes”. If there was sufficient evidence that CEUS and CT results were interpreted independently, items 10 and 11 were scored as “yes”. Clinical data such as history of trauma, patient demographics were available during the study and clinical practice, thus item 12 was scored “yes” for all individual studies. If no intermediate/uninterpretable test results were reported, item 13 was scored as “no”. If both test results were obtained, item 14 was scored as “no” for the individual study^17^.

**Table 1:** The QUADAS tool items and their corresponding numbers.

Item numbers	Items
1	Was the spectrum of patients representative of the patients with blunt abdominal trauma?
2	Were selection criteria clearly described?
3	Is the reference standard likely to correctly classify the target condition?
4	Is the time period between CT and CEUS short enough to be reasonably sure that the target condition did not change between the two tests?
5	Did the whole sample or a random selection of the sample, receive verification using a reference standard of diagnosis?
6	Did patients receive the same reference standard regardless of the index test result?
7	Was the reference standard independent of the index test (i.e. the index test did not form part of the reference standard)?
8	Was the execution of the index test described in sufficient detail to permit replication of the test?
9	Was the execution of the reference standard described in sufficient detail to permit its replication?
10	Were the index test results interpreted without knowledge of the results of the reference standard?
11	Were the reference standard results interpreted without knowledge of the results of the index test?
12	Were the same clinical data available when test results were interpreted as would be available when the test is used in practice?
13	Were uninterpretable/intermediate test results reported?
14	Were withdrawals from the study explained?

### Synthesis of results

The sensitivity and specificity are two interrelated quantities commonly reported for diagnostic test. Bivariate approach to the meta-analysis of diagnostic accuracy was employed in the study^18^. Quantities such as sensitivity, false positive rate and area under curve (AUC), as well as associated 95% confidence intervals were reported. Diagnostic odds ratio (DOR) was also reported for component studies, as well as for the pooled result. In analogy to meta-analysis of odds ratio (OR), meta-analysis of DOR was performed according to the method developed by Glas and colleagues^19^. Cochran’s Q statistic and Higgins I^2^ were reported for the assessment of heterogeneity of included studies^20^. A forest plot of the log(DOR) values together with the summary estimate was also presented. Subgroup analysis was performed by restricting dataset to the type of solid organs (e,g, spleen, liver and kidney). All statistical analyses were performed using R (version 3.3.2)^21^.

## Results

### Study selection

**Figure 1:**
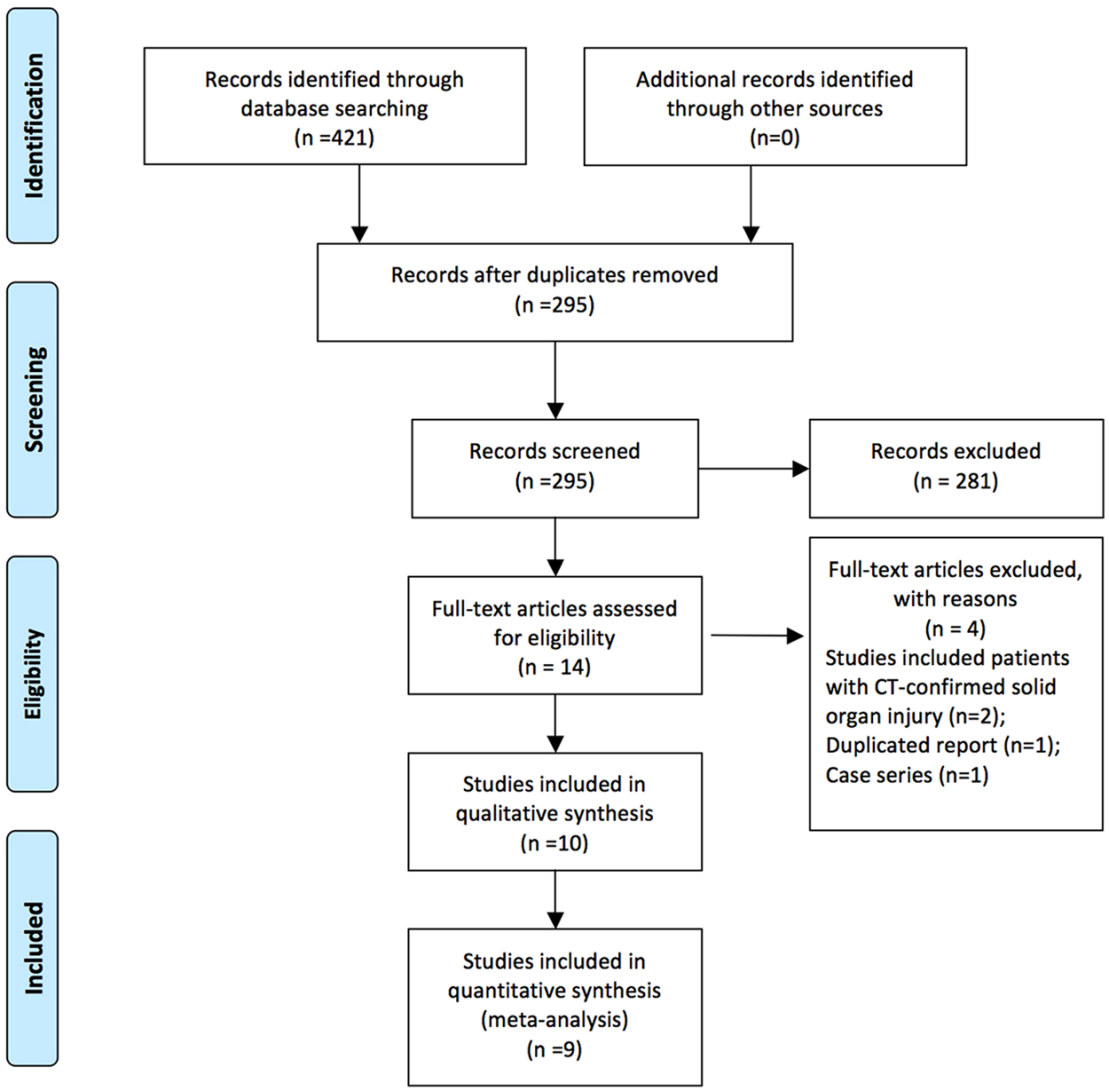
PRISMA flow chart of study inclusion. The initial search identified 421 citations. After removing duplicates, there remained 295 citations. A number of 281 citations were excluded, and the remaining 14 citations were reviewed for the full text. Four citations were excluded because two studies included only patients with CT-confirmed solid organ injury, one was duplicated report, and one is a case series report. As a result, a total of 10 studies were included in the study and 9 were included for meta-analysis.

The initial search identified 421 citations (Fig. 1). After removing duplicates, there remained 295 citations. A number of 281 citations were excluded because they were experimental studies, reviews, commentaries, case reports, and non-trauma conditions. The remaining 14 citations were reviewed for the full text. Four citations were excluded because two studies included only patients with CT-confirmed solid organ injury^22, 23^, one was duplicated report^16^, and one is a case series report^24^. As a result, a total of 10 studies were included in the study and 9 were included for meta-analysis^14, 15, 25–32^.

### Study characteristics

Characteristics of included studies are shown in Table 2. All studies were published after the year 2000, and five studies were published after 2010. The sample sizes of included studies ranged from 22 to 392. Four studies utilized prospective design, four studies employed retrospective design and the remaining two did not explicitly report the design. All studies included blunt abdominal trauma and the severity of trauma was mild to moderate. They used different words to describe the severity such as “low energy”, “minor”, “isolated” and “hemodynamically stable”. Most studies investigated the three major solid organs including kidney, spleen and liver. One study investigated diagnostic accuracy of CEUS on adrenals and found that CEUS missed two adrenal lesions as compared with CT^14^. Valentino’s study included patients with blunt abdominal trauma in which they among other injuries identified lesions in the pancreas and adrenals^29^. The included studies reported a wide range in the study participants’ age. One study included pediatric patients^26^, and two studies included both adult and pediatric patients^14, 15^. Three studies did not report summary statistics for the age^28–30^. Timing of CEUS was reported in two studies^14, 27^, and the remaining studies did not explicitly report the timing of CEUS. All studies reported the CT as reference standard, against which CEUS was compared.

**Table 2:** Characteristics of included studies.

Studies	Design	Sample size	Population	Sites	Age (years)	Experience of the operator	UCA (type/dose/No. injection)	Timing of CEUS	Reference
Miele 2016	Retro.	77	Blunt abdo. trauma	Kidney, spleen, liver, adrenals	8–61	Radiologist > 5 years’ experience	Sonovue/2.4 ml/2	24, 72 hrs and 1 months	CT
Menichini 2015	Retro.	73	Minor blunt abdo. trauma	Kidney, spleen, liver	8.7 ± 2.8	Radiologist > 10 years’ experience	Sonovue/1.2 ml/2	NR.	CT
Sessa 2015	Retro.	256	low-energy isolated abdominal trauma	Kidney, spleen, liver	7–82	Radiologist > 5 years’ experience	Sonovue/2.4 ml/2	NR.	CT
Lv 2011	Retro.	392	Liver or/and spleen trauma	Liver, spleen	NR.	Radiologist > 5 years’ experience	Sonovue/0.025 ml per kilogram/1	NR.	CT
Dormagen 2011	Pro.	22	Splenic embolization for trauma	Spleen	32 (15–57)	Radiologist with 8 and 10 years’ experience	Sonovue/2.4 ml/1	Prior to discharge; 3–4 months after discharge	CT
Valentino 2010	Pro.	133	Hemodynamically stable, blunt trauma	Kidney, spleen, liver, adrenals, pancrea	NR.	NR.	NR./2.4 ml/2	NR.	CT
Catalano 2009	Pro.	156	Blunt abdo. trauma	Kidney, spleen, liver	39 ± 17	NR.	Sonovue/2.4 ml/2	NR.	CT
Clevert 2008	Pro.	78	Blunt abdo. trauma	Kidney, spleen, liver	Mean:56	NR.	Sonovue/1.2–2.4 ml/1	NR.	CT
Miele 2004	NR.	203	Isolated abdo. trauma	Liver	36 (6–72)	NR.	Sonovue/NR./NR.	NR.	CT
*Catalano* 2003	NR.	25	Suspected abdo. injury that required CEUS	Spleen	NR.	NR.	Sonovue/4.8 ml/1	NR.	CT

Abbreviations: Retro.: retrospective; Pro.: prospective; NR.: not reported; CT: computed tomography; CEUS: contrast enhanced ultrasound; abdo.: abdominal.

### Risk of bias for individual study

The risk of bias was assessed using the QUADAS tool (Table 3). One study may introduce spectrum bias because it investigated parenchymal lesions after splenic embolization^27^. A substantial number of studies did not explicitly report how the results of CT and CEUS were interpreted, thus we could not exclude the possibility that the interpretation of the two tests influenced each other. In all studies, there were no special techniques required to interpret CEUS and CT results. Thus we considered that the same clinical data were available when CEUS were interpreted as would be available when CEUS is used in practice. No study reported uninterpretable/intermediate test results. No studies reported that patients were withdrawn from the study before the results of either or both of the CEUS and CT was known.

**Table 3:** Quality assessment of included studies using QUADAS tools.

Studies/Items	1	2	3	4	5	6	7	8	9	10	11	12	13	14
Miele 2016	Y	Y	Y	Y	Y	Y	Y	Y	Y	U	U	Y	N	N
Menichini 2015	Y	Y	Y	Y	Y	Y	Y	Y	Y	Y	Y	Y	N	N
Sessa 2015	Y	Y	Y	Y	Y	Y	Y	Y	Y	U	U	Y	N	N
Lv 2011	Y	Y	Y	Y	Y	Y	Y	Y	Y	U	U	Y	N	N
Dormagen 2011	N	Y	Y	Y	Y	Y	Y	Y	Y	U	Y	Y	N	N
Valentino 2010	Y	Y	Y	Y	Y	Y	Y	Y	Y	Y	U	Y	N	N
Catalano 2009	Y	Y	Y	Y	Y	Y	Y	Y	Y	Y	U	Y	N	N
Clevert 2008	Y	Y	Y	Y	Y	Y	Y	Y	Y	U	U	Y	N	N
Miele 2004	Y	Y	Y	Y	Y	Y	Y	Y	Y	U	U	Y	N	N
*Catalano* 2003	Y	Y	Y	Y	Y	Y	Y	Y	Y	U	U	Y	N	N

### Synthesis of results

**Figure 2:**
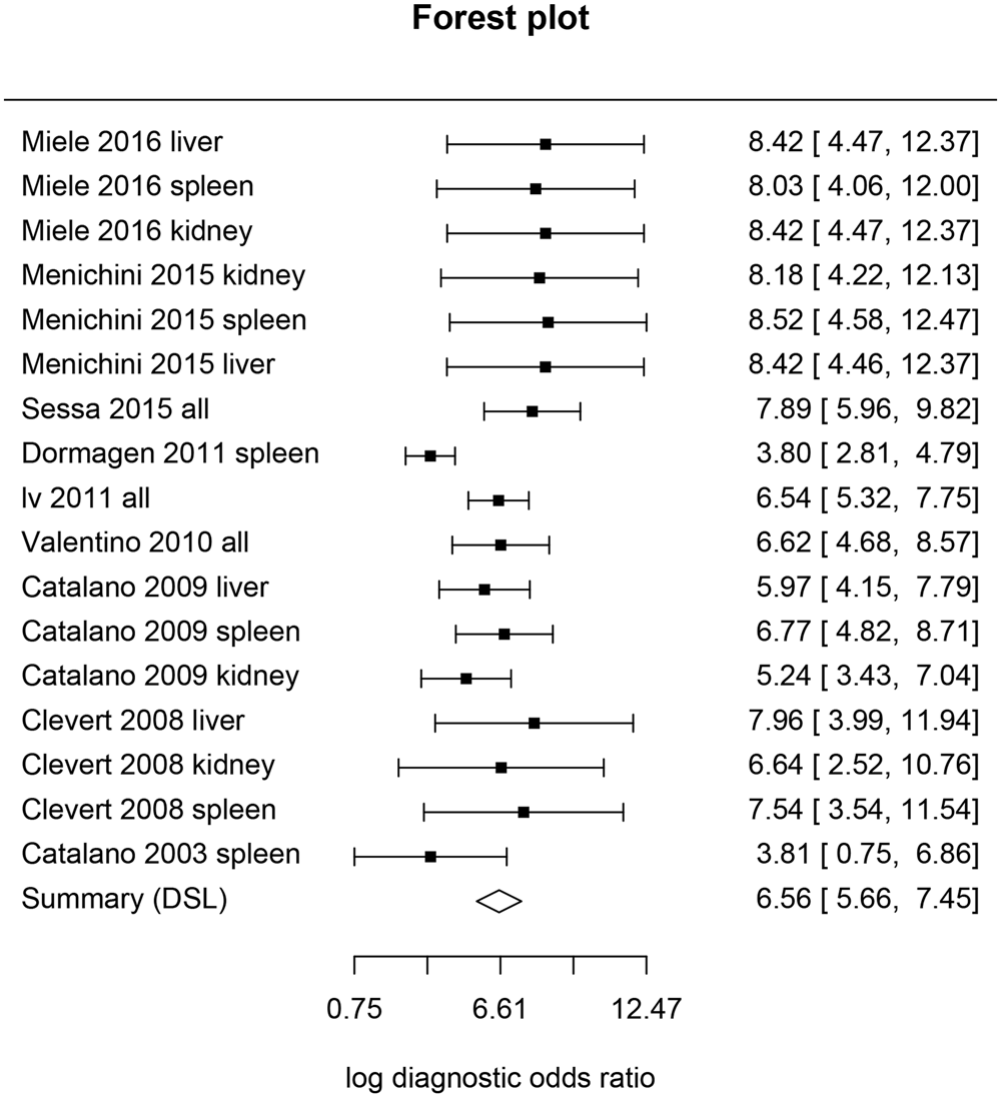
Forest plot showing log (diagnostic odds ratio [DOR]) for individual studies and summary estimate. The result showed that log(DOR) values ranged from 3.80 (95% CI: 2.81–4.79) to 8.52 (95% CI: 4.58–12.47). The combined log(DOR) was 6.56 (95% CI: 5.66–7.45). The Cochran’s Q was 11.265 (p = 0.793 with 16 degrees of freedom), and the Higgins’ I^2^ was 0%.

Log diagnostic odds ratio for individual study as well as the summary estimates are shown in Fig. 2. The log(DOR) values ranged from 3.80 (95% CI: 2.81–4.79) to 8.52 (95% CI: 4.58–12.47). The combined log(DOR) was 6.56 (95% CI: 5.66–7.45). The Cochran’s Q was 11.265 (p = 0.793 with 16 degrees of freedom), and the Higgins’ I^2^ was 0%.

**Figure 3:**
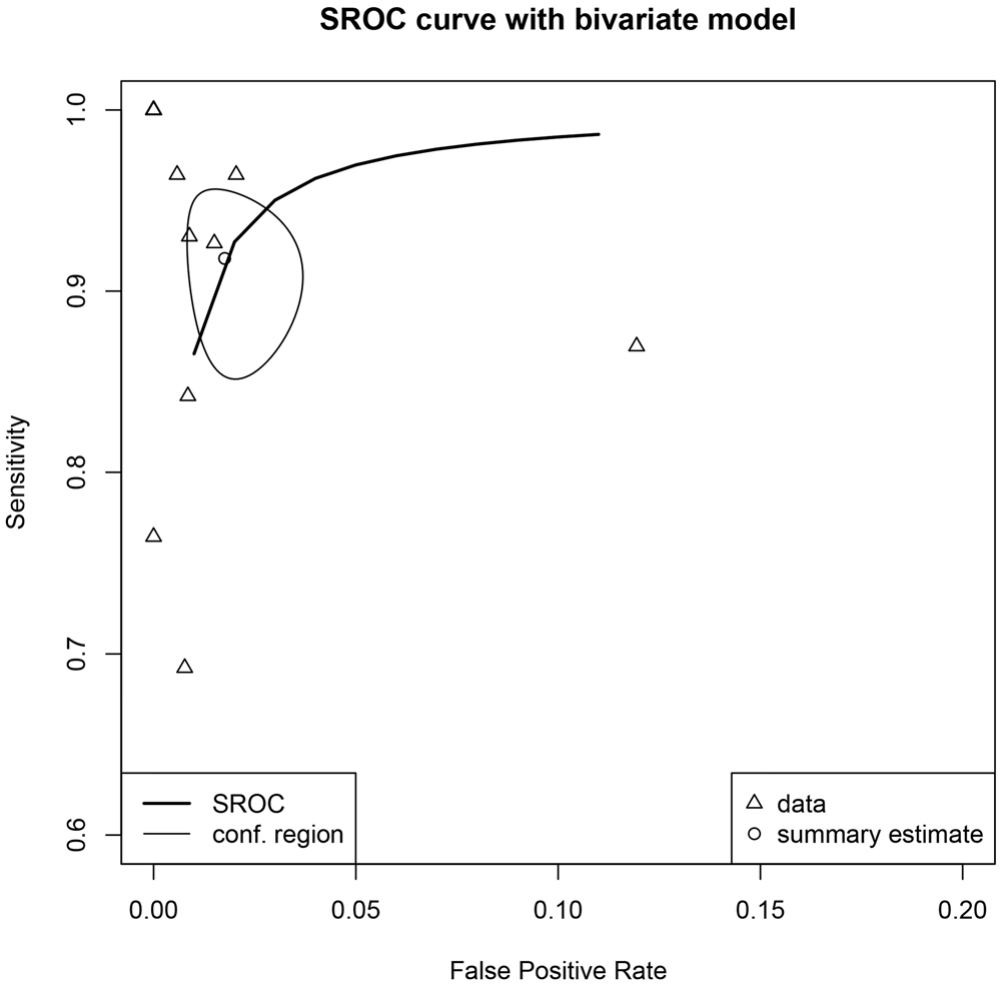
Summary receiver operating characteristic (SROC) curve plotting sensitivity against false positive rate (1-specificity). The summary estimate was calculated using bivariate approach. The result showed that the CEUS had a sensitivity of 0.981 (95% CI: 0.868–0.950) and a false positive rate of 0.018 (95% CI: 0.010–0.032) for identifying parenchymal injuries.

**Figure 4:**
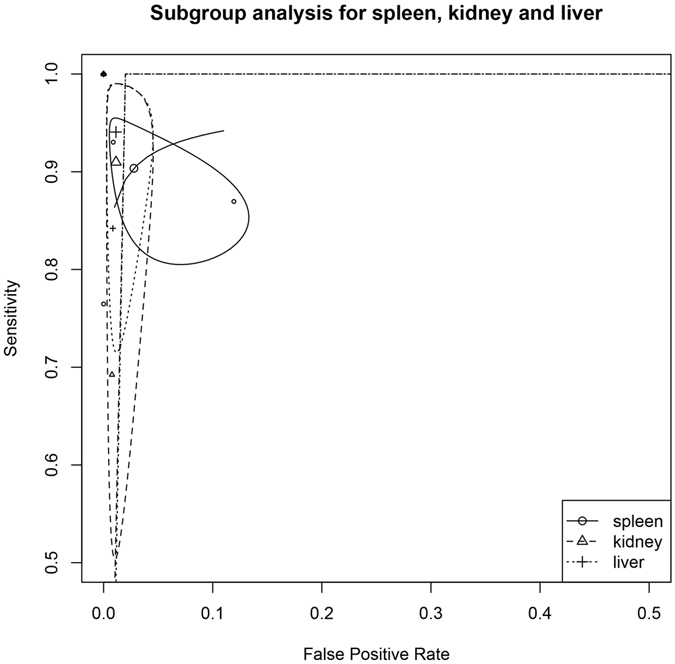
Summary receiver operating characteristic (SROC) curve for subgroup analysis restricting to different solid organs liver, kidney and spleen.

Pooled analysis of diagnostic accuracy of CEUS was performed using the bivariate approach. The included studies provided 17 datasets for pooled analysis. Overall, the CEUS had a sensitivity of 0.981 (95% CI: 0.868–0.950) and a false positive rate of 0.018 (95% CI: 0.010–0.032) for identifying parenchymal injuries (Table 4). The area under curve was 0.984. In subgroup analysis restricting to the type of solid organs the diagnostic accuracy of CEUS was excellent. The sensitivity and false positive rate for spleen were 0.904 (95% CI: 0.829–0.947) and 0.028 (95% CI: 0.007–0.099), respectively. The sensitivity and false positive rate for liver were 0.941 (95% CI: 0.784–0.986) and 0.011 (95% CI: 0.004–0.035), respectively. The sensitivity and false positive rate for kidney were 0.910 (95% CI: 0.616–0.984) and 0.011 (95% CI: 0.004–0.035), respectively. The summary receiver operating characteristic curves (SROC) were shown in Fig. 3 and subgroup analysis was shown in Fig. 4.

**Table 4:** Diagnostic performance of CEUS using computed tomography as reference standard.

Groups and subgroups	Sensitivity	95% CI	False positive rate	95% CI	AUC
All (n = 17)	0.981	0.868–0.950	0.018	0.010–0.032	0.984
Spleen (n = 6)	0.904	0.829–0.947	0.028	0.007–0.099	0.958
Liver (n = 4)	0.941	0.784–0.986	0.011	0.004–0.035	0.987
Kidney (n = 4)	0.910	0.616–0.984	0.011	0.004–0.035	0.987

Statistics were pooled using bivariate model. The n value in the first column was the number of datasets available for combination, and thus one study could provide more than one dataset.

## Discussion

The systematic review included 10 studies, 9 of which were included for meta-analysis. All studies reported good diagnostic accuracy of CEUS in identifying parenchymal lesions in patients with blunt abdominal trauma. There is no evidence of significant heterogeneity among included studies and among different types of solid organs (I^2^ = 0%). The result showed that the CEUS had a sensitivity of 0.981 (95% CI: 0.868–0.950) and a false positive rate of 0.018 (95% CI: 0.010–0.032) for identifying parenchymal injuries, and the area under curve was 0.984. The patients who are candidates for examination with CEUS are the hemodynamically stable patients with a history of low-energy blunt abdominal trauma. Since the diagnostic accuracy of CEUS is comparable to the CT scan, it is reasonable to recommend CEUS for assessment of patients presenting to the emergency department with a history of blunt abdominal trauma. Furthermore, CEUS is radiation-free and is suitable for repeated evaluations. For blunt abdominal trauma, delayed hemorrhage is common and thus repeated evaluations with CEUS is valuable in excluding potentially life-threatening conditions. Particularly, since there is trend shifting from operative to non-operative management of blunt splenic trauma, continuous monitoring of the lesion is of paramount importance^33^. CEUS provides a convenient and useful tool for such a purpose^13^.

However, CEUS has limited value in evaluating deep and small organs such as adrenals. In Miele’s study, all adrenal lesions were missed during CEUS, but they were confirmed with CT scan^14^. At one month follow up, the adrenal lesions were again missed in CEUS examination, but they could be identified by magnetic resonance imaging (MRI). Valentino’s study also reported that CEUS did not identify a lesion of the right kidney and an adrenal hematoma, which were later identified with CT^29^. For pancreas, it is reported that CEUS was able to identify 21/22 blunt pancreatic injury, yielding a false negative rate of 4.5%. However, this study only included patients with CT-confirmed pancreatic injury without reporting the false positive number, failing to provide a whole picture of the diagnostic accuracy^22^. Valentino M and colleagues reported a case of pancreatic injury. While B-mode US failed to demonstrate the injury lesion which was recognized by CT and MRI, CEUS well demonstrated the injury and was useful for monitoring during follow up of the lesion^34^. Although current evidence showed a good diagnostic performance of CEUS in identifying pancreatic lesions, the quality of evidence is limited. Lv’s study was retrospective in design that it was largely unknown whether the interpretation of CEUS result was influenced by CT findings or not^22^. In other words, the review bias could not be fully excluded^17^. As a result, the diagnostic accuracy of CEUS on pancreatic injury needs to be further examined in well design studies.

One limitation of the study was that the sample sizes of component studies were generally small. Half of the studies had a sample size below 100, which were traditionally regarded as small studies. There is evidence that small studies may overestimate the effect size in intervention meta-analysis^35^. Although small study effect has not been fully investigated in meta-analysis of diagnostic test, the result of our study should be interpreted with caution. However, studies with large sample size also showed an excellent diagnostic accuracy of the CEUS in identifying solid organ injuries, providing robustness to our results. It should also be noted that many studies were retrospective in design, which means that there was no strict study protocol for the performance of CT and CEUS. The concern with such study design is that the results of CT and CEUS may interfere with each other. In real clinical practice, emergency physicians have access to all imaging information (e.g. including CT findings if available) of a patient and this can influence their interpretation of CEUS findings. The traditional explanatory trials have been criticized for its external validity, because they are typically performed in experienced centers with strict inclusion/exclusion criteria^36–38^. In contrast, pragmatic trials allow better generalization of the result to the real world setting. In our example, pragmatic design means that physicians are not blind to imaging results during the performance of CEUS, confounding the diagnostic accuracy of CEUS. It is interesting to investigate how the presence of the results of other imaging studies can influence the interpretation of CEUS findings. It should be acknowledged that B-mode US and CEUS both have limited value in a trauma setting when evaluating bowel or mesenteric lesions. The images cannot be obtained clearly with the interference of bowel gas. Furthermore, the marketed ultrasound contrast agents are not excreted renally and therefore lesions in the collecting ducts are not visualized and could be overlooked^39^.

In conclusion, the study showed that CEUS performed at emergency department had good diagnostic accuracy in identifying solid organ injuries. It is a radiation-free technique that can be considered in monitoring solid organ injuries, especially for patients managed with non-operative strategy.

### Data availability statement

All data were available in original articles.

## Electronic supplementary material

appendix E1

## References

[1] Miele V (2016). Contrast-enhanced ultrasound (CEUS) in blunt abdominal trauma. Br J Radiol.

[2] Behnood, H. R., Haddadi, M., Sirous, S., Ainy, E. & Rezaei, R. Medical Costs and Economic Production Losses Caused by Road Traffic Injuries in Iran. *Trauma Monthly* in press (2016).

[3] van Beeck EF, van Roijen L, Mackenbach JP (1997). Medical costs and economic production losses due to injuries in the Netherlands. J Trauma.

[4] Brun P-M (2014). Stay and play eFAST or scoop and run eFAST? That is the question!. Am J Emerg Med.

[5] Markowitz JE, Hwang JQ, Moore CL (2011). Development and validation of a web-based assessment tool for the extended focused assessment with sonography in trauma examination. J Ultrasound Med.

[6] Nicolau C, Ripollés T (2012). Contrast-enhanced ultrasound in abdominal imaging. Abdom Imaging.

[7] Cagini L (2013). Contrast enhanced ultrasound (CEUS) in blunt abdominal trauma. Crit Ultrasound J.

[8] Afaq A, Harvey C, Aldin Z, Leen E, Cosgrove D (2012). Contrast-enhanced ultrasound in abdominal trauma. Eur J Emerg Med.

[9] Omar A, Freeman S (2016). Contrast-enhanced ultrasound of the spleen. Ultrasound.

[10] Cokkinos D (2012). Contrast-enhanced ultrasound for imaging blunt abdominal trauma - indications, description of the technique and imaging review. Ultraschall Med.

[11] Pinto F, Miele V, Scaglione M, Pinto A (2014). The use of contrast-enhanced ultrasound in blunt abdominal trauma: advantages and limitations. Acta Radiol.

[12] Piscaglia, F. *et al*. The EFSUMB Guidelines and Recommendations on the Clinical Practice of Contrast Enhanced Ultrasound (CEUS): update 2011 on non-hepatic applications. in **33**, 33–59 (© Georg Thieme Verlag KG Stuttgart · New York, 2012). 10.1055/s-0031-1281676 21874631

[13] Nolsøe CP, Lorentzen T (2016). International guidelines for contrast-enhanced ultrasonography: ultrasound imaging in the new millennium. Ultrasonography.

[14] Miele V, Piccolo CL, Sessa B, Trinci M, Galluzzo M (2016). Comparison between MRI and CEUS in the follow-up of patients with blunt abdominal trauma managed conservatively. Radiol Med.

[15] Sessa B (2015). Blunt abdominal trauma: role of contrast-enhanced ultrasound (CEUS) in the detection and staging of abdominal traumatic lesions compared to US and CE-MDCT. Radiol Med.

[16] Valentino M (2009). Contrast-enhanced ultrasonography in blunt abdominal trauma: considerations after 5 years of experience. Radiol Med.

[17] Whiting P, Rutjes AWS, Reitsma JB, Bossuyt PMM, Kleijnen J (2003). The development of QUADAS: a tool for the quality assessment of studies of diagnostic accuracy included in systematic reviews. BMC Med Res Methodol.

[18] Rutter CM, Gatsonis CA (2001). A hierarchical regression approach to meta-analysis of diagnostic test accuracy evaluations. Stat Med.

[19] Glas AS, Lijmer JG, Prins MH, Bonsel GJ, Bossuyt PMM (2003). The diagnostic odds ratio: a single indicator of test performance. J Clin Epidemiol.

[20] Higgins JPT, Thompson SG, Deeks JJ, Altman DG (2003). Measuring inconsistency in meta-analyses. BMJ.

[21] Zhang Z (2015). Data management by using R: big data clinical research series. Ann Transl Med.

[22] Lv F (2014). Emergency contrast-enhanced ultrasonography for pancreatic injuries in blunt abdominal trauma. Radiol Med.

[23] Mihalik JE, Smith RS, Toevs CC, Putnam AT, Foster JE (2012). The use of contrast-enhanced ultrasound for the evaluation of solid abdominal organ injury in patients with blunt abdominal trauma. J Trauma Acute Care Surg.

[24] Manetta R (2009). Ultrasound enhanced with sulphur-hexafluoride-filled microbubbles agent (SonoVue) in the follow-up of mild liver and spleen trauma. Radiol Med.

[25] Miele V (2004). Contrast enhanced ultrasound with second generation contrast agent in traumatic liver lesions. Radiol Med.

[26] Menichini G, Sessa B, Trinci M, Galluzzo M, Miele V (2015). Accuracy of contrast-enhanced ultrasound (CEUS) in the identification and characterization of traumatic solid organ lesions in children: a retrospective comparison with baseline US and CE-MDCT. Radiol Med.

[27] Dormagen J (2011). Contrast-enhanced ultrasound of the injured spleen after embolization–comparison with computed tomography. Ultraschall Med.

[28] Lv F (2011). Contrast-enhanced ultrasound imaging of active bleeding associated with hepatic and splenic trauma. Radiol Med.

[29] Valentino M (2010). Contrast-enhanced US evaluation in patients with blunt abdominal trauma. J Ultrasound.

[30] Catalano O (2009). CEUS in abdominal trauma: multi-center study. Abdom Imaging.

[31] Weckbach S, Minaifar N, Clevert D-A, Stickel M, Reiser M (2008). Contrast-enhanced ultrasound versus MS-CT in blunt abdominal trauma. Clin. Hemorheol. Microcirc..

[32] Catalano O, Lobianco R, Sandomenico F, Siani A (2003). Splenic trauma: evaluation with contrast-specific sonography and a second-generation contrast medium: preliminary experience. J Ultrasound Med.

[33] El-Matbouly M (2016). Blunt splenic trauma: Assessment, management and outcomes. Surgeon.

[34] Valentino M (2006). Contrast-enhanced ultrasound in non-operative management of pancreatic injury in childhood. Pediatr Radiol.

[35] Zhang Z, Xu X, Ni H (2013). Small studies may overestimate the effect sizes in critical care meta-analyses: a meta-epidemiological study. Crit Care.

[36] Ford I, Norrie J (2016). Pragmatic Trials. N. Engl. J. Med..

[37] English M (2016). The need for pragmatic clinical trials in low and middle income settings - taking essential neonatal interventions delivered as part of inpatient care as an illustrative example. BMC Med.

[38] Chen, S. C. & Kim, S. Y. A framework for analysis of research risks and benefits to participants in standard of care pragmatic clinical trials. *Clin Trials*, doi:10.1177/1740774516656945 (2016). 10.1177/1740774516656945 PMC5133165 27365010

[39] Pinto F, Valentino M, Romanini L, Basilico R, Miele V (2015). The role of CEUS in the assessment of haemodynamically stable patients with blunt abdominal trauma. Radiol Med.

