# Supplementary material for: Diagnostic accuracy of contrast enhanced ultrasound in patients with blunt abdominal trauma presenting to the emergency department: a systematic review and meta-analysis
Source: Sci Rep. 2017 Jun 30;7:4446. doi: 10.1038/s41598-017-04779-2 (PMC5493732; doi:10.1038/s41598-017-04779-2)
Supplement: Supplementary file 1 — appendix E1 [file 41598_2017_4779_MOESM1_ESM.doc]

pubmed

((((Contrast-enhanced ultrasonography[Title/Abstract]) OR Contrast-enhanced ultrasound[Title/Abstract])) AND (((trauma[Title/Abstract]) OR traumatic[Title/Abstract]) OR injury[Title/Abstract])) AND ((((liver[Title/Abstract]) OR spleen[Title/Abstract]) OR abdominal[Title/Abstract]) OR kidney[Title/Abstract]) OR pancrea[Title/Abstract])

SCOPUS

( TITLE-ABS-KEY ( **"Contrast-enhanced ultrasonography"** )  OR  TITLE-ABS-KEY ( **"Contrast-enhanced ultrasound"** ) )  AND  ( TITLE-ABS-KEY ( **" trauma"** )  OR  TITLE-ABS-KEY ( **"injury"** )  OR  TITLE-ABS-KEY ( **"traumatic"** ) )  AND ( TITLE-ABS-KEY ( **" liver"** )  OR  TITLE-ABS-KEY ( **"spleen"** )  OR  TITLE-ABS-KEY ( **"pancrea"** )  OR  TITLE-ABS-KEY ( **"abdominal"** ) )
